# Are functional movement disorder phenotypes or age at onset correlated with perfectionism or history of abuse?

**DOI:** 10.1016/j.prdoa.2021.100099

**Published:** 2021-06-04

**Authors:** Raja Mehanna, Liang Zhu, Carla Bejjani

**Affiliations:** a Department of Neurology, University of Texas Health Science Center at Houston, 6410 Fannin St, Houston, TX 77030, USA; b Center of Clinical and Translational Science, University of Texas Health Science Center at Houston, 7000 Fannin St, Houston, TX 77030, USA; c Department of Psychiatry, Baylor College of Medicine, 1 Baylor Plaza, Houston, TX 77030, USA

**Keywords:** Functional movement disorders, Age at onset, Rigid perfectionism, Abuse, Psychogenic movement disorders

## Abstract

**Objective:**

To assess if the phenotype or age at onset of Functional Movement Disorders (FMD) vary as a function of presence of a perfectionism or history of abuse. Detecting such a potential association might help guide future research into the pathophysiology of FMD.

**Methods:**

Charts of all patients diagnosed with FMD by a movement disorder specialist using the commonly accepted clinical diagnostic criteria for FMD seen at a tertiary center over 8 years were reviewed. Data collected were sex, age at the onset of the first FMD, phenotype of the first predominant FMD, history of perfectionism and history of childhood abuse (physical, sexual, emotional, or neglect). Statistical analyses were performed as appropriate.

**Results:**

68 patients with FMD were identified from which 12 were excluded for incomplete documentation. 56 patients were included in the analysis, 43 (76.8%) were women, with average age at onset 41.5y (range 13–74.4). The most frequent predominant initial FMD phenotypes were tremor (39%), dystonia (20.3%) and gait disorders (20.3%).Perfectionism was reported in 30 (53.6%) patients and history of abuse in 27 (48.2%).There was no significant correlation between each of the FMD phenotypes and perfectionism or history of childhood abuse. There was also no correlation between the age at symptoms onset and perfectionism or history of abuse.

**Conclusion:**

We could not demonstrate a significant correlation between FMD phenotype or age at onset and perfectionist personality trait or history of abuse. Factors leading to the development of one specific FMD phenotype rather than another are still to be elucidated.

## Highlights

• Charts of 56 patients with Functional Movement Disorders (FMD) were reviewed.
• 30(53.6%) had perfectionism (P), 27(48.2%) had history of childhood abuse (CA).
• 4(7.1%) denied P, CA or any psychiatric comorbidity.
• No correlation was detected between each of the FMD phenotypes and P or CA.
• No correlation was detected between the age at symptoms onset and P or CA.

## 1 Introduction

Functional Movement Disorders (FMD) are a form of conversion disorders, defined as involuntary movement disorders that are not due to a known medical disorder and thought to be psychologically mediated. FMD can present as any type of hypokinetic or hyperkinetic abnormal movements, including tremor, dystonia, parkinsonism, abnormal gait, and myoclonus. History of abuse, female gender and psychiatric comorbidities have been historically considered key risk factors for conversion disorders [1]. However, a higher prevalence of childhood sexual abuse and borderline personality disorder in patients with paroxysmal non-epileptic seizures (PNES) compared to patients with functional weakness suggests that different conversion presentations might be possibly linked to different psychopathologies [2]. A cross sectional study comparing 59 FMD patients with 43 PNES patients and 26 healthy volunteers reported higher level of conscientiousness in patients with FMD compared to the other two groups [3]. Conscientiousness has been associated with self-oriented perfectionism (setting excessively high standards for oneself) and is the personality trait most closely tied to obsessive–compulsive personality disorders [3]. It was suggested that self-oriented perfectionism, in the context of specific life events, can play a role in the genesis of FMD [3]. However, there is no data on a possible correlation between specific phenotypes or age at onset of FMD on one hand, and perfectionism and/or history of childhood abuse on the other. Detecting such a potential association might help guide future research into the pathophysiology of FMD.

We reviewed the charts of all patients diagnosed with FMD by a movement disorder specialist at our tertiary academic center to assess if the phenotype of FMD varied as a function of history of childhood abuse and/or the presence of perfectionism, and if the age at onset of FMD was correlated to the presence of childhood abuse and/or perfectionism.

## 2 Materials and methods

We searched our database for charts of patients with various ICD-9 or ICD 10 codes for FMD (Suppl Table 1) and seen in our movement disorder division between January 1, 2011 and December 31, 2019. These charts were then individually reviewed by a movement disorder fellowship trained specialist (RM) to confirm the diagnosis using the commonly accepted clinical diagnostic criteria for FMD [4]. This study was approved by the institutional IRB (approval number HSC-MS-20-0092). Data collected were sex at birth; age at the onset of the first FMD; phenotype of the first FMD; history of perfectionism; history of child maltreatment and neglect problems including childhood sexual, physical, or psychological abuse, as well as neglect, hereby referred to as childhood abuse; and history of other psychiatric disorders. This data is collected routinely at our center during clinical visit for a FMD, without the use of standardized scales. For perfectionism, we ask patient and family members if the patient is perceived as excessively disciplined, very detail oriented, gives excessive importance to order, or sets excessively high standards for oneself. A positive answer to any of these questions is recorded as perfectionism in the patient’s chart, irrespective of it interfering with task completion or causing significant distress or dysfunction to the patient. While this definition of perfectionism might be vague, it is intended to have a high sensitivity in order to capture the maximum of patients who show perfectionism. For childhood abuse, we ask the patient about the presence of any form of childhood abuse (physical, sexual, emotional) or neglect and elaborate on it to confirm its presence. Patients were included in the study if they were diagnosed with FMD by a movement disorder specialist and all the information detailed above was available. For statistical analyses, Chi-square test or fisher’s exact test were used to compare categorical variables between two groups, and two-sample *t* test or Wilcoxon rank sum test were used for continuous variables. The p value was considered significant at p < 0.05. All analyses were performed using the SAS 9.4 software (Cary, NC).

## 3 Results

**Fig. 1:**
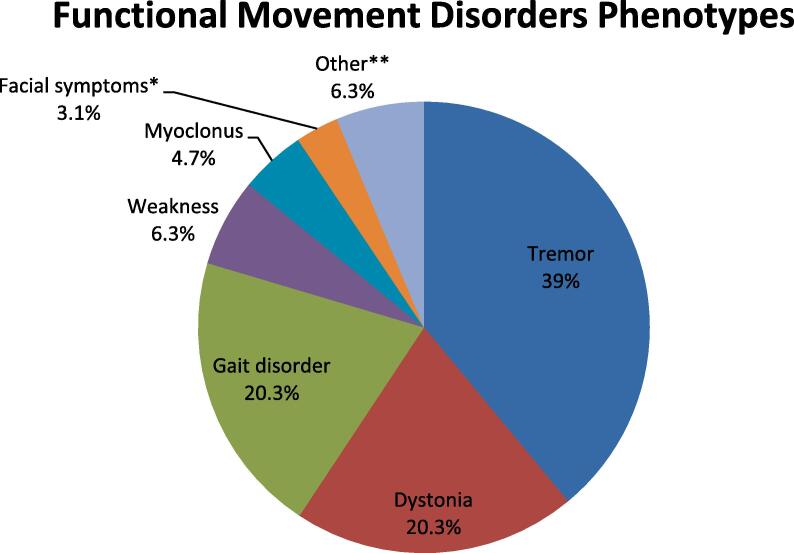
Functional movement disorders phenotypes. *These are patients who presented with symptoms limited to the face only, regardless of the phenotype. **This includes one patient with chorea, one patient with trunk and neck stereotypies, and two patients with tics.

A total of 68 patients with FMD diagnosed by a movement disorder specialist were identified, from which 12 were excluded for incomplete documentation. The remaining 56 patients were included in the analysis, with 43(76.8%) being women, and an average age at onset of 41.5 years (range 13–74.4). The most frequent predominant initial FMD phenotypes were tremor (39%), dystonia (20.3%) and gait disorders (20.3%) (Fig. 1). Perfectionism was reported in 30 (53.6%) patients and history of abuse in 27(48.2%), with 13 (22.2%) patients reporting both (Table 1). Among patients with perfectionism, 2 also had a history of depression and a third one had history of anxiety disorder. One patient with childhood abuse also had a bipolar disorder while another one had depression and panic attacks that preceded the abuse. Only 8 patients (14.3%) reported other psychiatric disorders but no perfectionism or history of abuse, including 3 patients with depression, 2 patients with bipolar disorder, 1 patient with anxiety, 1 patient with Asperger syndrome and 1 patient with schizoaffective disorder. Only 4 patients (7.1%) denied perfectionism, childhood abuse as well as any psychiatric comorbidity.

**Table 1:** Patients characteristics.

1. Age at onset (mean, range)	41.5y (13–74)
2. M/F	13/43
3. Patients with perfectionism (P)	30 (53.6%)
4. Patients with history of childhood abuse (CA)	27 (48.2%)
5. Patients with both P and CA (included in rows 3 and 4 above)	13 (22.2%)
6. Patients with other psychiatric comorbidities in addition to P or CA (included in rows 3 and 4 above)	5 (8.9%)
7. Patients without P or CA but with other psychiatric comorbidities.	8 (14.3%)
8. Patients without P, CA or any psychiatric comorbidity.	4 (7.1%)

There was no significant correlation between each of the primary FMD phenotypes and perfectionism or history of abuse (Suppl. Table 2). There was also no correlation between the age at symptoms’ onset and each of perfectionism (p = 0.31, two sample T test) or history of abuse (p = 0.42, Wilcoxon rank sum test).

## 4 Discussion

The majority of our patients (76.8%) were women, with an average age of onset of 41.5 years, which is similar to the published literature [3], [5], [6]. Similarly, the prevalence of the different phenotypes of FMD with tremor, dystonia and gait disorders representing the overwhelming majority of cases is in agreement with what was previously published [6]. Of note, while patients can have a fluctuation of the FMD phenotype overtime, we retained the dominant initial phenotype(s) for our study. More than 1 co-dominant phenotype was reported in 3 of our patients, and each phenotype was recorded and analyzed individually. There were thus a total of 64 recorded phenotypes in our 56 patients. Two patients were diagnosed with functional tics. While tics are notoriously difficult to distinguish from FMD because of their intermittent nature, fluctuation over time and improvement with distraction, the two patients reported here were strongly felt to have FMD by the movement disorder specialist who examined them in clinic.

Previous studies reported a higher rate of history of abuse in functional neurological disorders as a whole, compared with non–functional neurological disorders or healthy controls [7], as well as a significant association between sexual abuse and FMD in women [8]. While 48.2% of our patients reported a history of abuse, our series is the first to report on the prevalence of perfectionism in FMD, and we found it to be present in more than half our patients.

More broadly, our study shows that 92.9% of patient with FMD have an underlying psychiatric comorbidity, perfectionism and/or history of child abuse. One series of 28 FMD patients reported around 50% of psychiatric comorbidities, predominantly depression and anxiety [9]. However, it did not report on childhood abuse or pathological personality traits. Another series of 42 FMD patients reported lifetime major depression in 42.9%, anxiety disorders in 61.9% and categorical personality disorders in 45% [5]. However, it did not investigate a correlation between these psychiatric diagnoses and the FMD phenotype at presentation. It is important to note that while the presence of psychiatric comorbidities or history of childhood abuse may increase a neurologist’s certainty regarding the diagnosis of FMD, this is not a necessary criterion for the diagnosis, and many movement disorders specialists are comfortable making the diagnosis of FMD even in the absence of psycho-social dysfunction [4].

It has been suggested that different psychiatric disorders or psycho-social comorbidities could lead to different presentations of conversion disorders [2], [3], [4]. For example, a prospective study on 50 patients suggested that patients with PNES (n = 20) were more likely associated with a borderline personality disorders than patients with a motor conversion disorder namely weakness (n = 30) [2]. Another cross sectional study comparing 59 FMD patients with 43 PNES patients and 26 healthy volunteers reported higher level of conscientiousness – linked to self-centered perfectionism - in patients with FMD compared to the other two groups [3]. Understanding these differences may help with understanding why different semiology develops and could help with optimizing individualized treatment [3]. However, while 78.6% (n = 44) of our patients had perfectionism or history of childhood abuse, which could suggest a common underlying psychopathological process leading to FMD, our statistical analysis did not show any significant correlation between individual FMD phenotypes and perfectionism or history of abuse. The reasons or mechanisms leading to a specific phenotype in a given patient with FMD thus still need to be investigated. A possibility would be the presence of a family history of a specific movement disorder that would serve as a model for the FMD as reported in a few cases [10], [11], [12]. However, these cases are sparse in the literature and unlikely to completely explain the differential phenotypes in FMD.

Considering the potential role of perfectionism and history of childhood abuse in the pathogenesis of FMD, we also hypothesized that these might impact the age at onset differently and thus elucidate further the way each of these psychosocial comorbidities may impact the development of FMD. However, we could not find a correlation between the age at onset of the FMD and each of perfectionism or history of abuse and we are not aware of another study addressing this question.

Our study has several limitations. First, it is a retrospective chart review, which can raise concern about data collection and accuracy of diagnosis. However, all the patients were initially diagnosed and chart details were subsequently reviewed by fellowship trained movement disorder specialists, in order to ensure the accuracy of the diagnosis and completion of data. Charts with incomplete data were excluded to ensure the highest quality possible. Our study also lacks a control group, but our hypotheses were meant to detect correlations inside the FMD group, and did not require a control group. We also did not use formal tools to diagnose perfectionism or childhood abuse. While our definition of perfectionism might be vague, it was intended to have a high sensitivity in order to capture the maximum of patients who show any level of perfectionism. In addition, different authors support the use of different personality assessments, with some advocating quantitative measures such as the Revised Neuroticism-Extroversion-Openness Personality Inventory (NEO PI-R) [6],while other advocate categorical classifications [2], [5], contributing sometimes to conflicting results [3], [6]. Our systematic screening for perfectionism and childhood abuse in clinic could be perceived as a collection bias to the detriment of other mental health disorders and psycho-social conditions that were not screened for. However, the purpose of our study was not to compare the prevalence of different mental health disorders and psychosocial conditions in FMD, rather to look specifically at the prevalence and contribution of perfectionism and childhood abuse as these were suggested by others to potentially contribute to the development of FMD [3], [8]. One strength is that our sample size is similar to larger FMD series [3], [6] and larger than most others FMD series [5], [9], although our sample size may still not be sufficient to detect a difference between groups and could also contribute to the lack of correlation observed.

In conclusion, our study is the first to look at any potential correlation between specific phenotypes or age at onset of FMD on one hand and perfectionism and childhood abuse on the other. The lack of such correlations suggests that the factors leading to the development of one specific FMD phenotype rather than another are still to be elucidated. Larger studies using more objective psychiatric assessments are needed to confirm these results.

## Funding sources

None.

## 6 Disclosures

R Mehanna serves as a consultant for Global Kinetic Corporation, and is on the speaker bureau for TEVA, Adamas Pharmaceuticals, Acorda Therapeuthics, Sunovion and Kyowa Kirin. He has received consulting fees from Amneal and Sunovion, and research grants form Lundbeck, Global Kinetics Corporation, Acorda Therapeuthics, Prilenia Therapeutics and Solstice Neurosciences.

L Zhu has nothing to disclose.

C Bejjani has nothing to disclose.

## 7 Authors’ contribution

R Mehanna: conception and design, data collection, writing of the first manuscript, review and critique, editing, approval of the final version.

L Zhu: data analysis, review and critique, editing, approval of the final version

C Bejjani: review and critique, editing, approval of the final version

## CRediT authorship contribution statement

**R Mehanna:** Conceptualization, Data curation, Methodology, Supervision, Writing - original draft, Writing - review and editing. **L Zhu:** Formal analysis, Software, Writing - review and editing. **C Bejjani:** Writing - review and editing.

## Declaration of Competing Interest

The authors declare that they have no known competing financial interests or personal relationships that could have appeared to influence the work reported in this paper.

## References

[1] Thomas M., Jankovic J. (2004). Psychogenic movement disorders: diagnosis and management. CNS Drugs.

[2] Stone J., Sharpe M., Binzer M. (2004). Motor conversion symptoms and pseudoseizures: a comparison of clinical characteristics. Psychosomatics.

[3] Ekanayake V., Kranick S., LaFaver K., Naz A., Frank Webb A., LaFrance W.C., Hallett M., Voon V. (2017). Personality traits in psychogenic nonepileptic seizures (PNES) and psychogenic movement disorder (PMD): Neuroticism and perfectionism. J. Psychosom. Res..

[4] Espay A.J., Aybek S., Carson A., Edwards M.J., Goldstein L.H., Hallett M., LaFaver K., LaFrance W.C., Lang A.E., Nicholson T., Nielsen G., Reuber M., Voon V., Stone J., Morgante F. (2018). Current concepts in diagnosis and treatment of functional neurological disorders. JAMA Neurol..

[5] Feinstein A., Stergiopoulos V., Fine J., Lang A.E. (2001). Psychiatric outcome in patients with a psychogenic movement disorder: a prospective study. Neuropsychiatry Neuropsychol. Behav. Neurol..

[6] Kranick S., Ekanayake V., Martinez V., Ameli R., Hallett M., Voon V. (2011). Psychopathology and psychogenic movement disorders. Mov. Disord..

[7] Ludwig L., Pasman J.A., Nicholson T., Aybek S., David A.S., Tuck S., Kanaan R.A., Roelofs K., Carson A., Stone J. (2018). Stressful life events and maltreatment in conversion (functional neurological) disorder: systematic review and meta-analysis of case-control studies. Lancet Psychiatry.

[8] Kletenik I., Sillau S.H., Isfahani S.A., LaFaver K., Hallett M., Berman B.D. (2020). Gender as a risk factor for functional movement disorders: the role of sexual abuse. Mov Disord Clin Pract..

[9] Factor S.A., Podskalny G.D., Molho E.S. (1995). Psychogenic movement disorders: frequency, clinical profile, and characteristics. J. Neurol. Neurosurg. Psychiatry.

[10] Fahn S., Williams D.T. (1988). Psychogenic dystonia. Adv. Neurol..

[11] Fahn S., Sjaastad O. (1991). Hereditary essential myoclonus in a large Norwegian family. Mov. Disord..

[12] Fekete R., Jankovic J. (2010). Psychogenic chorea associated with family history of Huntington disease. Mov. Disord..

