# Supplementary material for: Are functional movement disorder phenotypes or age at onset correlated with perfectionism or history of abuse?
Source: Clin Park Relat Disord. 2021 Jun 4;4:100099. doi: 10.1016/j.prdoa.2021.100099 (PMC8299986; doi:10.1016/j.prdoa.2021.100099)
Supplement: Supplementary data 1 [file mmc1.docx]

Supplemental Table 1: ICD9 and IC 10 codes used to localize charts in our database.

| Name | ICD9 | ICD 10 |
| --- | --- | --- |
| Conversion disorder with abnormal movement | 300.11 | F44.4 |
| Functional movement disorder | 307.9 | F44.4 |
| Functional tremor | 306.0 | F45.8 |
| Psychogenic tremor | 306.0 | F44.4 |
| Functional gait disorder with tremor | 781.2  781.0 | R26.9  R25.1 |
| Functional gait abnormality | 781.2 | R26.89 |
| Functional gait disorder | 781.2 | R26.9 |
| Psychogenic gait | 307.9 | F44.4 |
| Dissociative ataxia | 307.9 | F44.4 |
| Hysterical ataxia | 300.11 | F44.4 |
| Astasia abasia | 307.9  300.11 | F44.4 |
| Psychogenic dystonia | 307.9 | F44.4 |
| Psychogenic torticollis | 306.0 | F45.8 |
| Functional dysphonia | 300.11 | F44.4 |
| Functional voice disorder | 784.49 | F44.4 |
| Psychogenic dysphonia | 306.1 | F44.4 |
| Psychogenic writer’s cramp | 300.89 | F48.8 |
| Psychogenic myoclonus | 333.2 | G25.3 |
| Functional chorea | 333.5 | G25.5 |
| Functional parkinsonism | 332.0 | G20 |
| Psychogenic parkinsonism | 332.1 | G21.8 |

Supplemental Table 2: Correlation Between FMD Phenotype and Perfectionism (Table 2A) and Childhood Abuse (Table 2B) (chi-square or Fisher's exact test)

| **Table 2A** | **Perfectionism** | |  |
| --- | --- | --- | --- |
| **Phenotype** | **No** | **Yes** | **P value** |
| Tremor | 13 (50.0%) | 11 (36.7%) | 0.31 |
| Dystonia | 3 (11.5%) | 9 (30.0%) | 0.11 |
| Gait Disorder | 8 (30.8%) | 5 (16.7%) | 0.34 |
| Weakness | 2 (7.7%) | 2 (6.7%) | 1.00 |
| Myoclonus | 0 (0.0%) | 3 (10.0%) | 0.24 |
| Facial Symptoms | 2 (7.7%) | 0 (0.0%) | 0.21 |
| Other | 2 (7.7%) | 2 (6.7%) | 1.00 |

| **Table 2B** | **Childhood Abuse** | |  |
| --- | --- | --- | --- |
| **Phenotype** | **No** | **Yes** | **P value** |
| Tremor | 12 (41.4%) | 12 (44.4%) | 0.82 |
| Dystonia | 6 (20.7%) | 6 (22.2%) | 0.89 |
| Gait Disorder | 6 (20.7%) | 7 (25.9%) | 0.64 |
| Weakness | 2 (6.9%) | 2 (7.4%) | 1.00 |
| Myoclonus | 2 (6.9%) | 1 (3.7%) | 1.00 |
| Facial Symptoms | 2 (6.9%) | 0 (0.0%) | 0.49 |
| Other | 3 (10.3%) | 1 (3.7%) | 0.61 |
